# Placenta accréta chez une primipare ayant un remplacement valvulaire: succès d'embolisation et gestion d'anticoagulants

**DOI:** 10.11604/pamj.2014.19.306.5703

**Published:** 2014-11-21

**Authors:** Nezha Oudghiri, Youssef Dahbi

**Affiliations:** 1 Département d'Anesthésie Réanimation Obstétricale, Hôpital Maternité Souissi, Université Mohamed V, Rabat, Maroc

**Keywords:** Anticoagulants, embolisation d'hémostase, placenta accréta de la primipare, valvulaire, Anticoagulants, haemostatic embolization, placenta accreta in primipara, valvular

## Image en medicine

Patiente âgée de 36 ans, primipare ayant un remplacement valvulaire aortique sous antivitamine K, consulte aux urgences obstétricales pour menace d'accouchement prématuré à 26 semaines d'aménorrhées. L’échographie initiale a montré un fœtus vivant et un placenta antérieur. On a remplacé les anti vitamines K par énoxaparine à la dose de 0,6ml/12h et on a commencé la tocolyse par des inhibiteurs calciques. Au quatrième jour, échappant à la tocolyse, la patiente a accouché par voie basse d'un nouveauné souffrant qui est décédé quelques heures après sa naissance. Le post-partum s'est compliqué d'une inertie utérine nécessitant une révision ramenant des débris placentaires et nécessitant l'administration d'ocytocine, du méthylergométrine, du misoprostol et la transfusion par quatre culots globulaires. L'hémorragie étant contrôlée, l’énoxaparine a été reprise à la douzième heure après l'accouchement. Au troisième jour du postpartum on a constaté une reprise de saignement avec à l’échographie une image évoquant une rétention placentaire, l’énoxaparine a été arrêté pour 24heures pour réaliser une aspiration évacuatrice de la cavité utérine. Le contrôle échographique a montré la persistance d'une rétention placentaire d'où la réalisation d'une IRM pelvienne qui a mis en évidence un placenta accréta. Au septième jour la patiente a bénéficié d'une artériographie utérine avec embolisation d'hémostase. L’évolution a été marquée par l'arrêt du saignement au quinzième jour du post-partum. La patiente est sortie sous anti vitamine K après un contrôle échographique satisfaisant.

**Figure 1:**
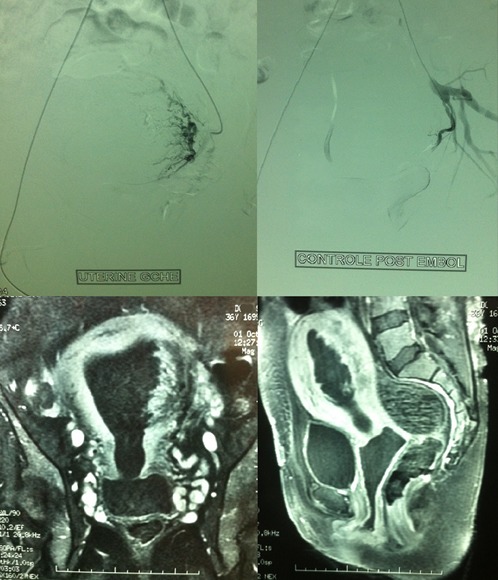
IRM diagnostiquant un placenta accréta chez une primipare et le succès de l'embolisation de l'artère utérine gauche

